# Six Collective Challenges for Sustainability of Almería Greenhouse Horticulture

**DOI:** 10.3390/ijerph16214097

**Published:** 2019-10-24

**Authors:** Antonio J. Castro, María D. López-Rodríguez, Cynthia Giagnocavo, Miguel Gimenez, Leticia Céspedes, Abel La Calle, Marisa Gallardo, Pablo Pumares, Javier Cabello, Estefanía Rodríguez, David Uclés, Salvador Parra, Jesús Casas, Francisco Rodríguez, Juan S. Fernandez-Prados, Daniela Alba-Patiño, Mónica Expósito-Granados, Beatriz E. Murillo-López, Lina M. Vasquez, Diego L. Valera

**Affiliations:** 1 Biology and Geology Department, Andalusian Center for the Assessment and Monitoring of Global Change (CAESCG), University of Almeria, La Cañada de San Urbano, 04120 Almería, Spain; leticespedes@gmail.com (L.C.); jcabello@ual.es (J.C.); jjcasas@ual.es (J.C.); fdaniela_alba@hotmail.com (D.A.-P.); moexposit@gmail.com (M.E.-G.); betymu@utp.edu.co (B.E.M.-L.); linamariavasquez7@gmail.com (L.M.V.); 2 Department of Biological Sciences, Idaho State University, 921 South 8th Avenue, Pocatello, ID 83209, USA; 3 Internet Interdisciplinary Institute (IN3)-Universitat Oberta de Catalunya (UOC), Av. Friedrich Gauss 5, 08860 Castelldefels, Barcelona, Spain; 4 Department of Economy and Business, Cátedra Coexphal-UAL Horticulture, Cooperative Studies and Sustainable Development, University of Almería, Agrifood Campus of International Excellence, CeiA3, and CIAMBITAL, 04120 Almería, Spain; miguel.gimenez@ual.es; 5 Department of Law, University of Almería, La Cañada de San Urbano, 04120 Almería, Spain; alacalle@ual.es; 6 Department of Engineering, CIAMBITAL Research Centre, University of Almería, Carretera de Sacramento s/n, La Cañada, 04120 Almería, Spaindvalera@ual.es (D.L.V.); 7 Department of Geography, History and Humanities, University of Almería, 04120 Almería, Spain; ppumares@ual.es (P.P.); jsprados@ual.es (J.S.F.-P.); 8 Centre for Migrations Studies and Intercultural Relations CEMyRI, University of Almería, 04120 Almería, Spain; 9 Instituto Investigación y Formación Agraria y Pesquera de Andalucía (IFAPA), Centro La Mojonera, 04745 Almeria, Spain; mestefania.rodriguez@juntadeandalucia.es (E.R.); salvador.parra@juntadeandalucia.es (S.P.); 10 Servicio de Estudios Agroalimentarios, Innovación Agroalimentaria, Cajamar, 04120 Almería, Spain; ducles@ual.es; 11 Departamento de Informática, Universidad de Almería, La Cañada de San Urbano, 04120 Almería, Spain; frrodrig@ual.es

**Keywords:** biodiversity, sustainable agriculture, circular economy, family farming, intensive agriculture, transdisciplinary science, knowledge transfer, governance, water

## Abstract

Globally, current food consumption and trade are placing unprecedented demand on agricultural systems and increasing pressure on natural resources, requiring tradeoffs between food security and environmental impacts especially given the tension between market-driven agriculture and agro-ecological goals. In order to illustrate the wicked social, economic and environmental challenges and processes to find transformative solutions, we focus on the largest concentration of greenhouses in the world located in the semi-arid coastal plain of South-east Spain. Almería family farming, predominantly cooperative, greenhouse intensive production, commenced after the 1960s and has resulted in very significant social and economic benefits for the region, while also having important negative environmental and biodiversity impacts, as well as creating new social challenges. The system currently finds itself in a crisis of diminishing economic benefits and increasing environmental and social dilemmas. Here, we present the outcomes of multi-actor, transdisciplinary research to review and provide collective insights for solutions-oriented research on the sustainability of Almeria’s agricultural sector. The multi-actor, transdisciplinary process implemented collectively, and supported by scientific literature, identified six fundamental challenges to transitioning to an agricultural model that aims to ameliorate risks and avoid a systemic collapse, whilst balancing a concern for profitability with sustainability: (1) Governance based on a culture of shared responsibility for sustainability, (2) Sustainable and efficient use of water, (3) Biodiversity conservation, (4) Implementing a circular economy plan, (5) Technology and knowledge transfer, and (6) Image and identity. We conclude that the multi-actor transdisciplinary approach successfully facilitated the creation of a culture of shared responsibility among public, private, academic, and civil society actors. Notwithstanding plural values, challenges and solutions identified by consensus point to a nascent acknowledgement of the strategic necessity to locate agricultural economic activity within social and environmental spheres.This paper demonstrates the need to establish transdisciplinary multi-actor work-schemes to continue collaboration and research for the transition to an agro-ecological model as a means to remain competitive and to create value.

## 1. Global Sustainability Challenges of Agricultural Systems

Human population growth and food consumption and waste are placing unprecedented demands on agricultural systems and increasing pressure on natural resources [1]. These demands are generating some of the most serious challenges faced by humanity [2], and are concurrently degrading land, water resources, biodiversity, and climate on a global scale [1,2,3]. Current predictions for the future of agriculture point out that to meet surging demand, global food production must double by 2050 [4]. In this sense, many authors note that to meet the world’s future food security and sustainability needs, food production must grow substantially while, at the same time, agriculture’s environmental footprint must shrink dramatically [2,3]. Despite the importance of addressing this global issue, it is receiving little urgency, and few quantitative targets are being developed by the global research and policy community [4].

Attaining sustainable global food systems implies, as a first step, understanding the trade-offs between food security (e.g., produce enough and healthy food) and environmental impacts (e.g., biodiversity loss, resource stresses) [3,5]. It has been extensively documented that impacts on biodiversity and natural resources are attributed to agricultural intensification (e.g., pesticides, fertilizers, tillage) and farmland abandonment (i.e. urbanization, job opportunities or aging populations). At a global scale, this environmental deterioration results in biodiversity loss and the decline of ecosystem services, placing the maintenance of human well-being at risk [6]. In this adverse scenario, new farming initiatives and renewed practices are urgently required to promote a sustainable agriculture model. This implies the transformation of intensive systems into more complex agroecosystems based on a recoupling of farming practices and their ecological and social environments [7]. Such farming transition should promote the adoption of farming initiatives and practices that preserve biodiversity, together with a bundle of agricultural-related ecosystem services such as pollination, erosion control, water management, cultural heritage and local identity [8,9,10,11]. To advance in this direction, agroecology has emerged as one of the most promising initiatives to support the transition from conventional to sustainable farming, improving both environmental sustainability (biodiversity and ecosystem services) and socio-economic conditions (food accessibility, participation and empowerment, and fair prices) [12]. However, to instigate such transition globally, a better understanding of the interactions between the social, economic and environmental dimensions of agricultural systems as well as the interdependencies and feedbacks between these dimensions is required [13].

In this paper, we consider an example of intensive greenhouse horticulture in order to explore the tradeoffs and challenges in bringing about a transition to a more sustainable agricultural production system. The paper is organized in five sections. In Section 1 we set out the background to the socio-economic-environmental challenges surrounding intensive greenhouse horticulture in Almería. Section 2 introduces the need for a new agro-ecological framework for sustainability. The transdisciplinary, multi-actor methodology is detailed in Section 3. Section 4 summarises the sustainability challenges identified in the workshop, and barriers and opportunities to address these challenges, and finally, actions to be taken. Section 5 sets out general conclusions and the importance of the six challenges identified herein as fundamental to transitioning into an agricultural model that ameliorates risks and avoids a systemic collapse, balances a concern for profitability with sustainability and provides crucial insights for the small family farming business model.

**Figure 1:**
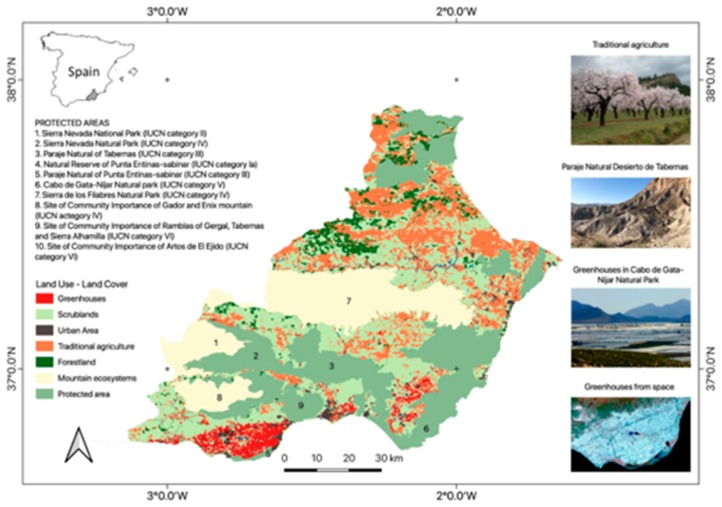
Geographic location of the greenhouse horticulture in Almeria, SE Spain.

In Europe, a significant part of the greenhouse horticulture is concentrated in the semi-arid coastal plain of the province of Almeria, in south-east Spain (Figure 1). The greenhouse horticulture production in Almeria started after the 1960s and currently houses the largest concentration of greenhouses in the world. Since 1960, development strategies and the lack of land use planning resulted in socio-economic development in coastal areas and caused one of the most dramatic land use transformations in Europe [14], currently representing around 4% of the provincial surface area. The promotion of greenhouse horticulture has resulted in very significant social and economic benefits for the Almería Province, while also having important negative impacts on native biodiversity and natural resources [15,16] as well as creating social challenges.

The economic contribution of greenhouse horticulture in Almería is approximately 1800 million Euros, and a related auxiliary business sector generates another 1.600 million Euros. In addition to the 15,000 family farmers engaged in production activity, 40,000 additional jobs are provided [17]. Within the province of Almería, greenhouse production represents 13% of gross domestic product (GDP), as contrasted to the average of agricultural GDP in Spain of 2.5% [18]. The total economic activity surrounding the farming system is 40% of the GDP of the province of Almería. However, this agricultural system has a relatively equitable distribution of wealth due to the fact that 95% of farms are family owned, and that their products are predominantly marketed by cooperatives or other social economy entities [17], as well as some investor-owned companies including a few auctions.

In Almería, small-holder family farm greenhouse horticulture started in earnest in the seventies, supported by local cooperative capital and knowledge transfer by way of the cooperative bank’s experimental farm, and the founding of marketing cooperatives as well as the association of horticultural exporters [19]. Greenhouse production is labour intensive and labour requirements were initially met by the local families of greenhouse owners. Since the end of the eighties, the increasing intensification of the family farming model has resulted in the need for family labour to be supported by immigrant labour, which mainly comes from different African countries [20] and Central-Eastern Europe. Currently, Almería’s greenhouse sector has over 110 nationalities working and requires several types of labour. Highly skilled and qualified workers that support these technological and innovative greenhouses [21] are necessary, as is manual unskilled labour, comprised of both family members and immigrant, who do the physical, routine work under difficult conditions inside the greenhouse [22]. In addition, within the cooperatives and producer organisations, the handling and packaging utilises a mostly female workforce which also represents a wide range of nationalities. As precision farming increasingly requires higher levels of technology and ICT knowledge, both lack of skills and investment in technology present social and economic challenges, not to mention the anticipated tensions that will be created by the mechanisation and automatisation of tasks traditionally held by workers.

While the economic benefits and both positive and negative social consequences have been evident due to the rapid and dramatic land transformation, greenhouse horticulture expansion has also produced significant impacts on local biodiversity and the decline of important ecosystem services (e.g., aquifer recharge or erosion control) [8,15,16]. The uniqueness of the biodiversity of arid and semiarid environments of the Almería region has been documented since the early nineties [23,24,25,26,27]. This region has been recently included among the 25 worldwide biodiversity hotspots and supports high levels of biodiversity, with numerous endemic species and habitats of priority interest at European levels [28,29,30,31]. This unique diversity of species and ecosystems has led to the declaration of diverse protected areas. However, in this region, these conservation efforts have co-existed and co-evolved with intense human developments (e.g., urban and agricultural expansion) over recent decades [32,33]. Historically, the conditions for human occupancy have been unfavourable, marked by scarce rainfall, rough land and frequent strong winds [34,35]. The human development model was fundamentally limited by water scarcity, and it was dedicated to subsistence dryland agriculture characterized by low yields [36]. As mentioned above, it was not until the 1970’s that this socio-economic model changed, led by the development of intensive agriculture, the tourism sector and the construction industry [14,36,37,38]. In particular, the rapid development of greenhouse agriculture along the Mediterranean coast has produced the alteration and fragmentation of habitat of numerous plant species and has affected the availability of groundwater resources [16,36]. As a consequence, simultaneously with these intensive land transformations, there has been a tremendous effort by governments to protect natural areas, especially those sensitive to the impacts of climate change and desertification [39]. As a result, several protected areas have been declared with different levels of protection status according to the International Union for Conservation of Nature (IUCN), including several new EU Sites of community importance of the “Natura-2000” network, the “Cabo de Gata Natural Park” in 1987 and the “Paraje Natural Desierto de Tabernas” in 1989 (Figure 1) [8,36,39]. The convergence of particular ecological and socio-economic factors in the region has led to conflicts of interest between conservation and human development [14].

The situation described above highlights the difficulty in determining tradeoffs and presents the “wicked” social and economic challenges implicit in making compatible market driven agriculture with agro-ecological goals. While the agricultural activity, largely based on social economy entities and family farms, has contributed greatly to the development of Almería and to the provision of healthy and safe food for Europe, serious social and environmental issues still persist, including informal contracting, lack of social integration and unregulated housing settlements, gender inequality, water aquifers/depletion and salinization, loss of biodiversity, and inadequate waste management (i.e., plastic and vegetable waste, fertilizers and pesticides). Whether small family farmers, their cooperatives and producer organizations, and related institutions will be in a position to carry out the necessary transformations in Almería in order to preserve positive social economic benefits, whilst meeting pressing agro-ecological challenges is an open question. We propose, as evidenced by this paper, that the academic community should play an important role in advancing solutions and closely work with all economic and social actors and local and regional administrations. Interactions between scientists and policymakers have often been limited to exchanges of scientific documents, and collaborative work between research and communities rarely occurs for addressing day-to-day social-economic-ecological problems. In this context, this paper is motivated by scientists concerned with aligning their research with real needs found in the local production system and the policy arena.

## 2. Transitioning to a Sustainable Model for the Almeria’s Agricultural Sector

**Figure 2:**
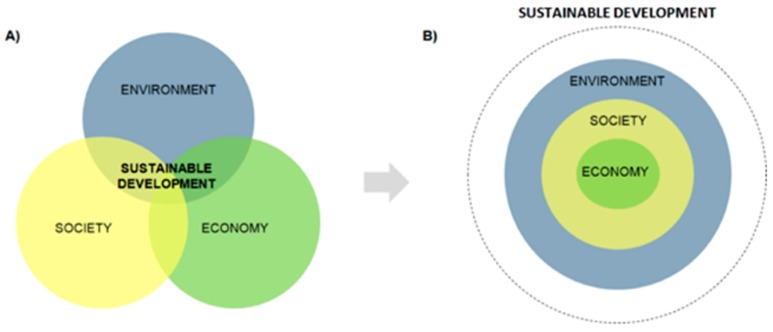
Conceptual framework of a sustainable agriculture model. Adapted from [42] (**A**) Three Pillars of Sustainable Development Model; (**B**) New Sustainable Development Conceptual Model.

There is a large body of literature that has advanced the conceptual models which contribute to understanding the basis of sustainability in socio-ecological systems (Figure 2) [40,41,42]. The Brundtland report “Our Common Future” [43] introduced the three pillars of sustainable development: economic, social, and environmental, which have been widely used especially in the policy arena (Figure 2A). In this approach, the three pillars are generally given equal weight and in many practical contexts sustainability is attributed to one of them at a time (e.g., sustainable economic growth) to the exclusion of the other two. There is a need to shift this paradigm into a new sustainability framework [6], recognizing the significance of the life-support systems and the biosphere as the foundation for the economy, society, and the human dimension. From such a perspective, it is explicit that the economy is a subsystem of society, which is, in turn, a subsystem of the biosphere or environment (Figure 2B) [44]. Within this sustainability framework, the long-term sustainability of greenhouse agriculture of Almería depends on the ability of the agricultural model to transition to a new sustainable framework which builds upon the biosphere as a precondition for socio-economic development [42]. Therefore, a major goal for Almeria’s greenhouse sector is to identify and understand the interactions and feedbacks among dynamic biophysical processes that regulate the biosphere (i.e., environment), and consequently its capacity for providing favourable conditions for complex human societies (i.e., society) and for long-term human prosperity (i.e., economy).

As in all other sectors of society, framing the agricultural sector in a new sustainability model based on recognition of the biosphere as the foundation upon which society and economy are based (Figure 2B) is extremely challenging. Specifically, the current socio-ecological context of Almeria’s agricultural sector illustrates complex realities resulting from balancing costs, benefits and trade-offs among social, economic and environmental dimensions that need to be analyzed with appropriate methods. For example, trade-offs are not straight forward when the inclusiveness of groups in processes would reduce cost effectiveness, or where business growth for one group of interest would lead to a loss in biodiversity affecting the common good [45]. On the other hand, tradeoffs which favor increased biodiversity and protection of the environment may lead to the loss of traditional jobs or businesses. In carrying out our analysis of the challenges that face the sustainability of Almeria’s agricultural sector, plural value-dimensions are present. These are often referred to as incommensurable values [46] as opposed to commensurable values, where in the latter, trade-offs are more straightforward to determine and solutions can be chosen by deciding which solution is optimal, using agreed upon values. In contrast, the presence of plural values when values cannot be compared by means of a single scale (e.g., monetary scale) means that the estimation of trade-offs is problematic [47]. An approach that allows for plural values to be considered is necessary in seeking solutions for the sustainability of Almería’s agricultural sector. In addition, stakeholder identification of unintended benefits or disadvantages are needed to avoid the risks of simplifying complexities and to fully clarify and include plural value dimensions, transparently identify trade-offs, and address the real drivers and barriers to future developments of Almeria’s agriculture sector. Therefore, an inclusive approach should take into account the development of common conceptual grounds through understanding and cooperation [48] in order to address divergence of values in the analysis of complex systems, where disagreements in definition or solutions of a societal problem often exist.

Coupled with this plural value and multiple criteria approach is the inclusion of multi-actors. The multi-actor approach is an adequate scientific response to stimulate cooperation of scientists with practitioners, industry and other actors. This approach can be implemented by using tools and procedures of transdisciplinary research). Transdisciplinary research combined with the multi-actor approach integrates knowledge from various scientific and societal bodies of knowledge [49] through co-learning and knowledge co-production processes [50] and contributes to solutions or transitions for societal challenges. Three key arguments justify the adoption of this approach. First, scientific disciplines are generally hyper-specific and represent a reductionist view to understanding the complexity and uncertainty of human nature systems [51]. Overcoming the barriers of such disciplinary specificity and fragmentation facilitates a holistic view of human nature interactions and allows one to combine and obtain different forms of knowledge to generate context-based knowledge [52]. Second, research on workable solutions by scientists requires an understanding of the socioeconomic, cultural and political-administrative context to align scientific information with policy processes and societal demands [53]. Finally, cooperation between academics and non-academics is helpful in terms of legitimacy, ownership and shared responsibility for implementing problem-solving solutions [54,55,56]. These approaches have been gradually increasing in multiple areas of sustainable governance such as water, agriculture and forests to help society transition towards sustainability [57,58,59]. However, multi-actor and transdisciplinary approaches remain underutilized for developing strategies and actions for the sustainability of Almeria greenhouse horticulture and recent research suggests the need for transdisciplinary science [29,34].

Below, we report the outcomes of research based on the implementation of a multi-actor, transdisciplinary approach and methodology, including an initial questionnaire, multi-actor participation and evaluation, all supported by transdisciplinary scientific research and literature. The workshop is intended to serve as a starting point to provide collective insights for solutions-oriented research on the sustainability of Almeria’s agricultural sector, in order to better identify and characterize challenges needed for long-term sustainability of the region.

## 3. Methodology: A Transdisciplinary Process for Identifying Sustainability Challenges

As part of the research methodology, a multi-actor structured workshop was conducted in April 2019 at the University of Almeria (Spain). The goal of the workshop was to collectively identify the key challenges for long-term sustainability of greenhouse horticulture of Almeria. A multi-actor community of practice was organized [60] that included scientists and non-scientists with extensive experience in greenhouse agriculture from five knowledge domains (i.e., technology, environment, society and culture, economy, and governance). Non-scientists included public and private actors, including but not limited to cooperative presidents, public extension services, foundations, farmers associations, etc. Considering time and financial constraints a total of 15 multi-actor experts (three within each domain) were invited to participate in the workshop. To select these experts the ‘reputational approach’ [61] was used, through which knowledgeable individuals in greenhouse agriculture in the study area were asked to select leading experts from diverse knowledge domains.

**Figure 3:**
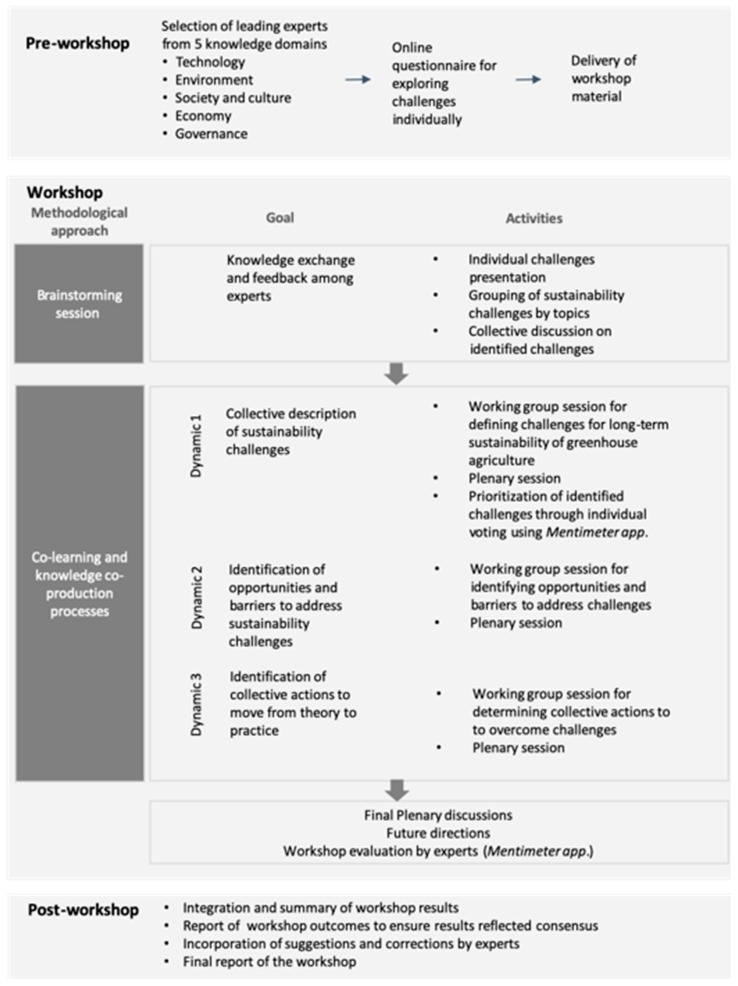
Methodological workflow of the workshop for addressing sustainability challenges for the greenhouse horticulture in Almeria (Spain). Adapted from [33].

Prior to the structured workshop, the experts were invited to complete an online questionnaire (Figure 3) (Appendix A). The purpose of this questionnaire was to individually, based on a review from different transdisciplinary expertise, identify three key challenges limiting sustainability of Almeria greenhouse agriculture. To ensure operability in the workshop, all experts were supplied with a timetable, goals pursued in the research, work schemes, and a full list of participants (Appendix B). The workshop was facilitated by two researchers with extensive experience and skills in multi-actor, transdisciplinary science. Both promoted knowledge transfer between the experts and facilitated communication and understanding between them [62]. At the beginning of the workshop, presentations were given by the facilitators to provide a deeper understanding of the rationale, goals and methods of the workshop (Figure 3). Thereafter, each expert explained the challenges that she/he had identified from an individual perspective in the online questionnaire. All individual challenges were grouped according to their similarity by the facilitators using a card-writing system. By and through the brainstorming on challenges, the experts started to enrich their perspectives from other knowledge domains.

Thereafter, three collective dynamics were conducted (Figure 3). Methodologically, these dynamics were based on co-learning and knowledge co-production approaches in order to generate outcomes based on a balance of trade-offs among the experts from diverse knowledge domains [54]. The collective dynamics were oriented specifically to (1) explore and define the sustainability challenges, (2) identify opportunities and barriers to address each challenge, and (3) establish collective actions to move from theory to practice in overcoming the identified challenges. All the collective dynamics shared the same structure: (1) introduction by the facilitators to explain goals and rules to develop the activity, (2) co-generation of outcomes through a multi-actor work group with mixed profiles, and (3) exposition of results by each work group in plenary to generate feedback and synergies between team groups. Each workgroup involved multi-actor experts of each knowledge domain to promote an integrated view of the sustainability of greenhouse agriculture from multiple perspectives. Each workgroup had a technical secretary who took minutes of the meeting. Upon completion of the three collective dynamics, all co-generated outcomes were collectively discussed and future directions to address the key challenges were identified by the experts. Throughout the dynamics the Mentimeter online application (https://www.mentimeter.com/) was used by the experts in order to prioritize the sustainability challenges and evaluate the workshop. This online software application allowed participants to vote in real-time and reach a consensus on the results. This provided transparency and credibility to the process. Once the workshop results were compiled by the facilitators, they were sent to the participants for their suggestions and corrections, ensuring that the results reflected consensus.

## 4. Sustainability Challenges for the Spanish Greenhouse Horticulture

**Figure 4:**
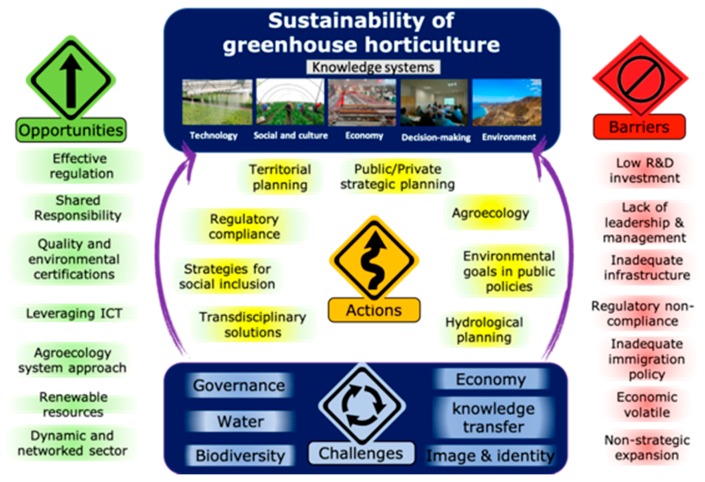
Sustainability challenges, opportunities, barriers and actions for the sustainability of the greenhouse horticulture.

As a result of the structured workshop methodology, participants collectively identified six challenges for ensuring the sustainability of the greenhouse horticulture in southeast Spain (Figure 4); The challenges corresponded to (1) Governance based on a culture of shared responsibility for sustainability, (2) Sustainable and efficient use of water, (3) Biodiversity conservation, (4) Implementing a circular economy plan, (5) Technology and knowledge transfer, and (6) Image and identity. Each challenge is introduced below, with its context and rationale, opportunities and barriers, related supporting scientific literature, and key collective actions to address it.

### 4.1. Sustainability Challenge 1: Governance Based on a Culture of Shared Responsibility for Sustainability

The first sustainability challenge describes the need to shift to a new governance system that enables the Almería greenhouse horticulture to evolve towards a culture of shared responsibility for its long-term sustainability. A major governance issue of Almería agricultural sector identified in the workshop is the regulatory non-compliance tolerated by public local and regional administrations. It was observed that such non-compliance may benefit small farmers in the short term but harm Almería’s society, including farmers, in the long term. We use the term “governance” in a rather limited sense in this article to refer to the overall aim to exercise public power (i.e., rules, procedures and practices) with the goal of deepening democracy through the active and direct participation of society in decision-making. In the context of environmental sustainability literature, governance refers to the set of regulatory processes, mechanisms and organizations through which political actors influence environmental actions and outcomes [63]. According to [64], in the past, the role of civil society in creating public policies and governance was neglected. According to the United Nations Development Program [65], governance is defined as the set of mechanisms, processes, relationships and institutions through which citizens and groups articulate their interests, exercise their rights and obligations, and reconcile their differences. In addition, it is important to understand the different ways in which society relates to the government to define public policies [66]. The diversity of governance interpretations and meanings, dependent as they are on plural values, as well as multidisciplinary traditions, results in a complex task in the attempt to define a common and generic governance strategy. As strategies for achieving adequate governance, the United Nations Development Programme and the European Union proposed key parameters that included promoting participation, making public decisions with transparency, building consensus on issues of general interest, the efficient use of public resources and accountability. This implies establishing a culture of shared responsibility through the involvement of all societal actors in decision making. In order to achieve this challenge, there is a need to facilitate new and practical forms of cooperation between governments and society, with a new view to carry out common tasks and negotiating and mobilizing coalitions of interest to achieve common purposes.

The analysis of the governance challenge provided the foundation for identifying opportunities and barriers to address it. Among the opportunities found was the existence of a legal framework that obligates public administrations to guarantee transparency and active participation in policies such as the Convenio de Aarhus 1998, Ley 26/2007, Real Decreto Legislativo 1/2001, Ley 19/2013 and Ley 1/2014, or the legal framework that requires economic agents to assume corporate social responsibility (Law 18/2018) and environmental responsibility (Law 26/2007). Among the barriers identified was the fact that the public administration has not developed an effective method by which to fulfil the obligation to include active public participation. In doing so, it is crucial to empower Almeria’s society by offering training and experience in public participation for public policies, which requires that governments increase their legitimacy through greater engagement. More recently, the European Commission has emphasized the full engagement of public participation in governance and achievement of its research missions, where the public is not only involved in co-creation but also in co-implementation and co-evaluation [67].

On this basis, a key action to advance in a new governance model for the agricultural sector in Almeria is to improve internal and inter-institutional coordination for the integration of environmental goals in public policies. Moving to sustainable governance model requires the creation of new spaces based on social learning and collective construction for sustainability, instead of the negotiation of individual interests and preferences [68]. For instance, the creation of public policies, at all levels, which acknowledge the creation of environmental goods and account for their value, which would benefit small farmers and society in general. By doing so, the economic risk of transitioning to a more sustainable agriculture would be compensated and not borne solely by small farmers, who may not be in a position to financially weather such change in the short term. Farmers could be seen to also be producers of environmental goods, and thus should be rewarded for contributing to sustainability. It was recently suggested to follow the protocol of the report of the Sustainable Development Goals 2030 ODS 17 “Partnership to Achieve the Goals”, which states that a successful sustainable development program requires partnerships between governments, the private sector and society. These alliances must be inclusive, built on common principles and values, and a shared vision that understands that Almería’s agricultural system must keep growth within the biophysical boundaries. Also, the costs and efforts of implementing such vision must be equitably distributed.

### 4.2. Sustainability Challenge 2: Sustainable and Efficient Use of Water

Unsustainable water management is one of the most important environmental issues worldwide in the twenty-first century [69]. This global issue is especially relevant in Mediterranean semi-arid ecosystems of south-east Spain, where the scarcity and unpredictability of rainfall events and the lack of perennial rivers mean that water recharged by aquifer systems is the main source of water resources for irrigation and consumption. In addition, groundwater water resources play a key role not only in the conservation of unique ecosystems but also in sustaining the wellbeing of local communities through the supply of ecosystem services [70]. This challenge corresponded to the urgent need of correcting the current trend of water use by greenhouse horticulture, which has caused over the last forty years severe deterioration of water quality and quantity [8,16]. In particular, the main water issues associated to the greenhouse horticulture are: (a) aquifer overexploitation, which corresponds to about 80% of water sources for irrigation, and whose water deficit is currently estimated for the Campo de Dalias in 65 Hm^3^ year^−1^; (b) water quality deterioration due to a progressive increase of water salinity in aquifers, which causes damage and losses of saline-sensitive horticultural crops. Water quality deterioration is caused by two major forces; marine intrusion processes and an unsustainable aquifer management [16,71]; and (c) the nitrate (NO_3_^−^) contamination of underlying aquifers and wetlands caused by the use of fertilizers the greenhouse-based vegetable production system [22]. Much of the area where greenhouses are located has been declared Nitrate Vulnerable Zones (NVZ) in accordance with the Nitrate Directive of the European Union, which requires the adoption of improved management of both irrigation and nitrogen [72,73].

The integrated use of alternative water sources from desalination, purification and rain harvesting and groundwater with the appropriate blending system to optimize cost, quality and water extraction rates can help to reduce the overexploitation of the aquifers [22]. Several Almería farmers, cooperatives and associations are doing so and taking action. With the aim of achieving the sustainable and efficient use of water resources, diverse water authorities from regional to national scale (i.e., Andalusian Water Agency and ACUAMED), in coordination with the Poniente Water User’s Board and the Geological and Mining Institute of Spain, launched the program for support the regeneration and recuperation of the aquifer system of Sur de Sierra de Gádor-Campo de Dalías in 2008. This program is based on a combination of measures and alternative water sources to restore the body of groundwater with the aim of achieving gradually its good status within 12 years. Whereas this plan has represented a successful initiative of coordination and joint efforts among governmental, societal and scientific entities, there remains uncertainty whether the planned goal will be achieved. In the relation to the higher costs of this alternative water source and according to [74], the incentives promoting the use of desalinated seawater for irrigation that most encourage farmers would be the implementation of tax relief, price reductions and the obligation to install rainwater collection systems.

Within this context, several opportunities for addressing this challenge were found. Such opportunities include (1) the compliance of the EU Water Framework Directive, which requires public administrations and economic agents to cooperate to achieve the good condition of all water bodies; (2) the existence of low-cost technologies developed and currently evaluated in the greenhouse industry as result of innovative research and knowledge transfer that would imply significant water savings and reduced nitrate leaching (see, for instance, the Fertinnowa project, https://www.fertinnowa.com/project/, the Internet of Food and Farm IoF2020, chain integrated greenhouse vegetable trial https://www.iof2020.eu/trials/vegetables/chain-integrated-greenhouse-production, the water and nutrient hubs established under peer-to-peer learning EU project NEFERTITI https://nefertiti-h2020.eu, and the recent founding of the Almería SmartAgriHub by the University of Almería, COEXPHAL and the Cajamar Foundation); (3) the existence of a very effective system for knowledge dissemination to greenhouse farmers, cooperatives and supply companies where the dissemination is conducted through technical advising by public and private organizations such as the Cajamar Foundation, COEXPHAL the Almería Association of F&V Producer, the Instituto de Investigación y Formación Agraria y Pesquera de Andalucía IFAPA, and the Centro de Investigación en Biotecnología Agroalimentaria (BITAL) at the University of Almería; (4) the existence of a market that is increasingly demanding more sustainable production processes, and which can be achieved by different certification systems (for example, GLOBAL GAP good agricultural practices certification); and (5) a very flexible and dynamic community of farmers and cooperatives capable of absorbing changes and promoting new strategies in the greenhouse industry. Barriers identified include the following: (1) a lack of public awareness by water users regarding the urgent need of a sustainable use of water resources and the implications for the long term sustainability of the greenhouse industry; (2) reticence by water users to pay for higher-cost irrigation water from alternatives strategies; (3) a lack of economic incentives by public administrations to change the current water management plans; and (4) a lack of control by public administrations over existing water regulations to prohibit illegal water practices such as the use of illegal wells.

Finally, actions identified amongst the participants include the following: (1) to adapt and control water use rights according to availability (Water Exploitation Index) and progress towards compliance with the water and regulatory framework in force (water use for agriculture and nitrates); (2) to provide the sector with regular information on the general condition of the groundwater bodies and create a panel of experts including both socio-economic agents (companies, unions, etc.) and the civil society (consumers, environmental organisations); (3) to promote the use of alternative water sources and efficient and sustainable irrigation and fertilisation management systems; and (4) to ensure the impact of the transfer of technologies and knowledge from local R&D organizations.

### 4.3. Sustainability Challenge 3: Biodiversity Conservation

The challenge of biodiversity conservation not only describes the need for protecting one of the most unique habitats of Europe, i.e., the Spanish drylands, but also the urgent need to communicate to society the interdependent relationship between the maintenance of the greenhouse horticulture and conservation of local natural resources [32,34]. Although Almería greenhouses only occupies 4% of the province of Almeria, most are concentrated in the coastal platform where numerous habitats of priority interest are located [8]. For instance, in the southern part of the coastal platform called “El Campo de Dalias”, there are different Sites of Community Importance of the European Natura 2000 network, e.g., Paraje Natural of Punta Entinas-Sabinar (IUCN category III) and Natural Reserve of Punta Entinas-Sabinar (IUCN category Ia) (Figure 1), that protect priority habitats and species of community interest that contribute to maintenance of biodiversity (e.g., bird species) and ecosystem services (e.g., erosion control and aquifer recharge) [39]. However, major changes occurred in recent decades in the economic activities of the Almeria province have led to severe impacts on these habitats. Among these habitats are the community of *Ziziphus* (i.e., Priority Habitat 5220, Directive 92/43/ECC), one of the priority habitats for conservation in Europe that has almost disappeared [15,75] due to the expansion of greenhouse industry, urbanization and beach tourism.

Among the opportunities to address this challenge, participants identified (1) the existence of local regulations that oblige the establishment of green infrastructures in the surroundings of new creation greenhouses (B.O.P. of Almería number 148 of 3 August 2017); and (2) the existence of incentives by regional administration to subsidize the establishment of green infrastructures (BOJA number 69 of 11 April 2017). In addition, new green infrastructures will be supported by the Strategy for Green Infrastructure and Ecological Connectivity and Restoration [76]; (3) the dynamic and adaptive character of Almería’s greenhouse industry, which shows a permanent capacity and flexibility to implement new measures and innovations [14,77]; (4) new technologies and cultivation techniques to reduce harmful agricultural practices, such as integrated pest management, organic production, improved irrigation, decision support systems, internet of things, and digitisation in general could contribute to support biodiversity; and (5) better communication with consumers to promote sustainable agricultural products. Among the barriers, we found (1) the poor perception by all levels of society of the value of biodiversity and the direct and indirect benefits it provides to people, such as erosion control, maintenance of coastal and beach dynamics, flash-flooding control or the existence value of a unique biodiversity in Europe [11,78]; (2) the restoration plant species of interest (e.g., *Maytenus senegalensis* subsp. *europaea* and *Ziziphus lotus*, *Rosmarinus officinalis* and *Thymelaea hirsuta*) is constrained by the greenhouse expansion; and (3) the globalised competitive horticultural markets which prioritise high, short-term economic performance, and the focus on technology to increase production at all costs. The latter has led to a socio-ecological vicious circle in which natural ecosystems are transformed into agricultural spaces that demand increasingly stressed ecosystem services to maintain their production.

Among the actions identified we found (1) increased public understanding of the need to preserve ecological processes that sustain the greenhouse industry (e.g., planting of autochthonous hedges between greenhouses); (2) implementation of effective measures for acknowledging the instrumental values of biodiversity. Examples of this instrumental value include for instance the crucial role of Sierra de Gádor mountain does in recharging aquifers that sustain the greenhouse industry and the importance of native flora species in the maintenance of insects for biological control [79,80,81,82]; and (3) the restoration of local ephemeral streams (i.e., ramblas in Spanish) which will contribute to the maintenance of genetic diversity, and the habitat connectivity of the Nature 2000 Network. All these measures can contribute to ensuring the proper conservation of Natura 2000 sites in the region, as a basis for the population’s understanding of the importance of conservation policies, and compliance with environmental regulations.

### 4.4. Sustainability Challenge 4: Implementing a Circular Economy Plan

The European Commission launched the Action Plan for promoting the Circular Economy in 2015. Subsequently, in 2018, both the Spanish and Andalusian governments adapted and designed their own circular economy plan. At the regional level, the government introduced circular bioeconomy as an “economic model based on the use of renewable biomass resources and their sustainable and efficient transformation into bioproducts, bioenergy and services for society”. In this sense, the Almería production model lacks the non-closure of its material circuits. On one hand, plastics (both packaging and those used to protect crops) are a serious problem for which the solutions so far have been to use regulated disposal companies, who either recycle or transform into energy, or burn the plastics which cannot be recycled or utilised otherwise. On the other hand, accumulated crop residues represent a logistical, environmental and health issue, and are not yet solved, as there are not sufficient facilities which deal with this waste in a circular manner. Furthermore, leachate resulting from agricultural irrigation can end up in aquifers, worsening the quality of underground water resources.

A relevant opportunity was identified in the growing societal awareness associated with sustainability issues in general, and among consumers in particular. In this sense, the existence in Almería of a local productive system could facilitate the transfer of values, knowledge and technologies [83]. In addition, the economic capital from agricultural industry surpluses and other sectors such as tourism, construction and industry could bring together the development of new sectors. Finally, the wide availability of sunlight in Almeria offers a parallel opportunity to the agricultural industry associated with new photovoltaic plant businesses [84]. Among the obstacles that could hinder this challenge are the logistical difficulties related to the small farms agricultural model, where waste collection and recycling is complex and expensive. The sector is disadvantaged by a low level of research, development and innovation and research investment by the agricultural industry [85].

Key actions to achieve this challenge include the following: (1) the implementation by regional administration of promised initiatives that facilitate the sustainable use of resources by the agricultural industry; (2) the creation of knowledge and agricultural innovation system that facilitates sustainable strategies among all relevant public and private actors; (3) increased investment in research, development and innovation (R + D + i), through sustainable finance initiatives, venture capital and networks of business angles that invest in finding solutions for the local productive system; and (4) support for existing R + D + i initiatives between the research and business sectors, including but not limited to initiatives between the university and cooperative finance, the Almería SmartAgriHub, various operating groups that have been set up under EIP Agri to specifically focus on water use, plastics, vegetable waste, phytosanitary products, etc.

### 4.5. Sustainability Challenge 5: Technology and Knowledge Transfer

In general, agricultural value chains are being transformed by digital technologies. The Almería agricultural sector is highly dependent on the use of adequate technologies adapted for Mediterranean greenhouse systems, yet it lags behind in adoption. Increasing consolidation and concentration in the agribusiness sector, particularly in the seed, fertiliser, chemical, genetics, and farm machinery industries, has been evident for decades, and it is expected that the new data technologies will drive the trend even more so [86]. The retail sector is concentrated among five or six major actors. As a result, Almería farmers and farming community SMEs risk being squeezed in the middle between digitally savvy and powerful input suppliers and buyers and stuck in a production-oriented paradigm within traditional, sequential supply chains, where intermediaries enjoy the surplus value created. In addition, while agri-food supply chains and value ecosystems are converting into data-rich and data-defined sectors, the value of farmers’ data is often unrealized by the farming community, and data ownership and control issues are neglected. Finally, while technologies and digitization may present opportunities to solve a range of agricultural challenges, including increasing sustainability, alternatively, they may create new social problems, inequalities and exclusion, extending the “power of the powerful” and allowing socio-economically advantaged actors, who are well connected and highly skilled, to scale up rapidly, and extend their social, economic, cultural and geographic influence [87]. Successfully adopting appropriate technologies, knowledge systems and organisational approaches to address these factors and generate positive outcomes and mitigate negative impacts will determine whether Almería agriculture will become more sustainable and remain competitive in an increasingly globalized agri-food market.

Beginning with the Almería production process, the workshop experts noted that the introduction of technology in the greenhouse has two basic objectives related to sustainability: (1) to maximize production, improve quality and minimize the use of resources and improve their management (i.e., water, electricity, labor), (2) technologies are utilised for the integration of information from different sources that help the producer to make decisions or to implement decision-support systems in production processes: irrigation, climate, disease management, planning of workers’ tasks, etc. Such technologies may also be used to improve and monitor regulatory compliance (e.g., nitrates, pesticides, water use, etc.).

While the greenhouse production system is implicitly connected to technologies, in Almería it may still be considered to be a “mid-tech” system. Marketing cooperatives have more sophisticated technology in place in their handling facilities particularly due to the demands of complying with strict food safety, traceability and certification requirements, and the intricacies of dealing with complex supply chains and exports, not to mention the requirements of complying with EU regulations pertaining to producer organizations. Yet at the greenhouse level, most farms are either low-tech or mid-tech. Less than 10% of farms utilise basic sensors which could contribute to increased efficiency in water use or control of climate conditions.

The results of the workshop indicated that the adoption of technology was seen to be most related to increasing economic benefits for the farmers, their cooperatives and also the surrounding auxiliary businesses, but such adoption was also seen to have an important role in making Almería agriculture more sustainable from an environmental point of view, with possibilities for social benefits as well. In the case of Almería, barriers to successful transformation include: (1) technologies are still in their early stages and untested so that applying them to the cooperatives’ business ecosystem remains complex. Of particular concern is the risk and uncertainty of adopting and implementing technologies and whether they will prove beneficial from a cost/benefit perspective. Also, there has been an explosion of technical and digital “solutions” and guidance is lacking as to credible and tested devices and systems; (2) the lack of data standards and interoperability between devices and systems; (3) people play a central role in digital technology and must adapt to the new working methods and skills required, particularly through the implementation of appropriate knowledge acquisition; (4) the laws and regulations applicable to data from the agricultural industry is a source of concern as it is unclear, insufficient and inconsistent; and (5) given its small-holding, family farming organizational structure, doubts arise as to whether the necessary investments may be made by such fragmented enterprises. With this in mind, experts found that technology adoption and digital transformation represented a significant challenge for farmers and cooperatives, but also a real opportunity to create value. Opportunities were found in the fact that technologies already exist which could be adopted and that producers had clear necessities to do so. Financing is available to a certain extent and examples of successful technology adoption and digitization already exist. Also, there has been an increase in public data made available (for example, climate and market data), thus providing the groundwork for innovations and allowing more effective solutions to be created.

In order to realise these opportunities, certain actions are required that mainly center around transfer of knowledge and technology, as well as training. Entities which can facilitate uptake and transformation in Almería include research and experimental centers, technology companies, cooperatives and the association of producer organisations (COEXPHAL) and a public administration concerned with knowledge transfer and uptake of technology and digitisation. Financing for such initiatives was also was seen to be available. Barriers to taking advantage of opportunities included the insufficient communication between business, university and producers, as well as the lack of technology for certain important agricultural tasks. The workshop participants also proposed that enterprises that generate and analyse data should come to an agreement on and elaborate data standards to exchange data and achieve interoperability. As a result, it was determined that Almería institutions such as research centers, technology companies, cooperatives and associations of producer organisations, as well as the administration, should collaborate and foster coordination in areas of research, whilst striving to improve transfer activities and create data standards. While technological innovation and the advancement of technology were seen to be important, the corresponding social innovation was seen to be equally important. Social innovation, which is closely aligned with processes of uptake of technologies, implies changes of attitudes, behaviours or perceptions within a network of people with “aligned interests that lead to new and improved ways of collaborative action within the group and beyond” [88]. Hence, technical and social innovation are closely tied to the socio-economic and ecosystem impact of technology advances in Almería. An organisational structure like that of cooperatives may prove to be a method to effectively and efficiently serve the farming community in an equitable and inclusive manner, and as an instrument to counteract the negative social and economic impacts of technology and digitisation of agriculture [89]. Priorities include closing the “digital divide” and addressing the “rural penalty” in ICT where coverage and broadband are often sub-optimum, de-risking adoption and experimentation through cooperative testing facilities or university research centers. Almeria’s cooperatives may support and provide a range of activities, from enhancing skills, encouraging adoption through a genuinely open approach to farmer needs and values, co-creation, and creating access through networks and platforms. Data generated by agricultural activities may be collected and analysed to provide valuable information to reduce environmental impacts. As part of the transformation, cooperatives may need to change their business models or create new cooperatives such as a data cooperative, to create economies of scale, and to function as coordination mechanisms. Their strategy should incorporate the opportunities for more sustainable practices that new tools have to offer.

### 4.6. Sustainability Challenge 6: Image and Identity

The challenge of improving the image and identity of Almeria’s agricultural sector is structured into three components, (1) the improvement of rural hygiene in the surroundings of the greenhouse area, (2) promoting socially just and fair labor conditions, and (3) the need to publicize and inform the international community of the commitment of the agricultural sector to move forward in a sustainable model of agriculture where the economy operates within the limits of a sustainable environmental system.

Almeria’s agricultural sector has shown in recent decades a great strength becoming an international recognised exporter of horticultural products. However, the urgent need to improve the visual impact and pollution produced by greenhouse horticulture is today a pending and urgent challenge. Examples of these impacts are, for example, the disturbing image of ephemeral streams (i.e., *ramblas* in Spanish) overflowing with the tide of garbage due to a deficient plan of rural hygiene, or the need to implement a management model for organic waste (e.g., plants) and inorganic waste (e.g., plastics). Recent research showed that the deterioration of the image has had an impact on the agricultural sector, including a reduction of demands among European consumers, ultimately leading to a decrease in imported goods [90], and affecting international consumers’ perception, which are today increasingly inclined to make informed purchasing decisions concerning horticultural products [91]. In addition, these findings indicate that European destination markets of the Spanish greenhouse horticulture could have a preconceived image; however, nationally the production system does not seem to influence the image of the same [91].

Another important issue related to the image of the agricultural sector is the need for fair labor conditions. Since conflicts concerning immigrants which occurred in 2000 in El Ejido [92,93,94,95,96,97,98,99], Almeria’s image deteriorated and was associated by national, and especially international, media with poor treatment and living conditions of migrant workers, therefore turning it into a subject-matter of debate due to work tensions, ethnicity and gender [94]. The rapid development of the agricultural sector favours the flow of migrants attracted by the need for labour [95,96]. In this way, the so-called “Almeria miracle” is necessarily associated with important demographic and labor market transformations in the province [14] and is based on the wage-earning work of immigrant workers. The demands of the Almeria agricultural labour market have mainly targeted and attracted migrants from Morocco and several West African countries whose living conditions have been defined by different authors as poor and precarious [97], with processes of labour segmentation [98], residential segregation [99], substandard housing [100] and the existence of scattered settlements [101,102]. Thus, social sustainability is questioned [103] despite the importance of family farms [104].

Finally, the image of the sector has also been affected by the negative impact of campaigns and news in both the national and international media on the Almería greenhouse model [90], mainly due to the sector’s lack of capacity to address existing problems. However, this research indicated that some of the crises in the past were the result of unfounded accusations [90]. All these challenges have burdened the image of Almeria’s agriculture internationally without a proactive attitude to solve it by the agricultural sector and public administrations. The public administrations have developed integration programmes, such as the Integral Plan for Immigration of the Andalusian regional government, Junta de Andalucía, implemented by local administrations [105], and have granted access to education and health public services, despite the legal situation of immigrants. However, the natives’ attitudes and discourses, the media and the emergence of a xenophobic political party in the province constitute barriers that diminishes the capacity of policies, responses or actions aimed to integrate at the full inclusion of the migrant collective, especially in sensitive issues as housing. The prevailing view in the agricultural sector, that many of those problems are not their responsibility or that they are too complex (both views were exposed during the workshop), has for long time hampered a deeper engagement to face this situation. Nevertheless, the growing importance of corporate social responsibility and the beginning of a demand by international distribution chains for controls on the living conditions of workers seems to be pushing a greater business awareness of these issues. Generalising and increasing the criteria of the social practice modules of certifications such as GRASP [106] provide an opportunity to improve working conditions and the image of the sector. Since its image is severely affected by these social issues, and since agriculture gives employment to more than half of legal foreign workers in the province and that they are the majority of their salaried workers, the sector should make an additional effort and play a more prominent role in encouraging or joining initiatives to promote social inclusion to clearly show its commitment to change.

Among the opportunities and barriers to address the above issues, workshop participants identified (1) the implementation of sanctions and creating the obligation for agrochemical companies to collect waste from their products and to pay for the packaging and recycling of each product, which will imply overcoming barriers related to costs and the need to convince private landowners; (2) improving the working and social conditions of immigrant workers by the eradication of shantytowns and the promotion of settlements with decent housing alternatives for seasonal workers, and the elaboration of an integration plan for immigrants, which implies knowledge of the staffing needs at each stage of the agricultural campaign [107]; and (3) improvement of the national and international image through new awareness campaigns financed with private funds of the agricultural sector, which would imply a change of mentality in the agricultural sector on the need to recognize the consequences of this issue and the need to invest in initiatives to change it. Finally, the actions identified by workshop participants included (1) new regulations on greenhouse structures and installations, the use of plant screens in greenhouses and awareness-raising campaigns; (2) a closer collaboration with local administrations and entities concerned with the plight of immigrants that have a long experience of intervention and social integration [108]; and (3) new local strategies, funded privately, to improve the image of the sector nationally and internationally. Identified actors who can play a pivotal role included the interprofessional entity HortyEspaña, the association of producer organisations, Coexphal, farm worker unions, and local and national authorities (given immigration is a national competence). Individual cooperatives also have a role to play, wherein leading and successful cooperatives do provide dignified housing and can serve as an example for others to follow suit.

## 5. Conclusions

While the transdisciplinary multi-actor research and workshop activities provided a wide range of challenges and proposed solutions, as described above, this research paper concludes with two main observations, having to do with both methodology and strategy. The multi-actor transdisciplinary approach implemented herein facilitated the collective work among scientists and nonscientists to progress in finding solutions to sustainability issues of Almería greenhouse horticulture and to find pathways for the transition to an agro-ecological model, creating a culture of shared responsibility among public, private, academic, and civil society actors. This study should be considered as a starting point to encourage iterative, open and ongoing processes which are embedded within institutional settings and that evolve and change over time by strengthening collaboration and building trust and common understanding among science, policy and society. Whereas it is assumed that the study provided insights arising from the particular case chosen as an example, it is widely recognized that such insights provide a useful contextual orientation to design further transdisciplinary experiences in other contexts and sectors.

The six challenges identified herein as fundamental to transitioning into an agricultural model that ameliorates risks and avoids a systemic collapse balance a concern for profitability with sustainability and provides crucial insights for the small family farming business model. Originally organised around cooperatives which were founded to create economies of scale and avoid “market failure”, Almería small farmers and their producer organisations have been attempting to survive and extract value according to the rules of the competitive, global agro-industrial business model, resulting in the aforementioned socio-ecological vicious circle based on an extractive logic. However, given decreasing benefits and increasing global competition which often depends on delocalized capital and production, the sector finds itself in crisis. Many of the challenges and opportunities identified herein are closely linked with finding added value in sustainability rather than in the standard market logic of efficiency (i.e., decreased inputs, increased outputs). The workshop represents a nascent awareness, notwithstanding the presence of plural values, of a new strategic business perspective: the necessity for the transition to an agro-ecological model in order to remain competitive and create value. Whereas as the agro-industrial model relies on the “corporate social responsibility” perspective to address environmental or social issues, the Almería model, embedded as it is within its territory and social environment (already largely based on the social economy of cooperative institutions), has the opportunity to realise a transition to a business model based on sustainability. However, this opportunity includes overcoming significant challenges, and the transition will by no means be an easy feat. Any solution will require collective action and significant innovation.

## Appendix A. Online Survey to Review and Identify Challenges of the Sustainability of Greenhouse Horticulture in Almeria, Spain

As reported, the objective of the workshop is to individually, based on a review from different transdisciplinary expertise, identify three key challenges limiting sustainability of Almerian greenhouse agriculture. In order to prepare the workshop effectively, we ask you to respond to this short questionnaire based on your experience, knowledge, and scientific evidences.

The results of all questionnaires completed by the participants will be used as initial input to develop the workshop.

Thank you very much for your collaboration.

1. Name and affiliation.
  ………………………………………………………………………………………………….………
2. Which is your motivation for participating in this workshop?
  ………………………………………………………………………………………………….………
3. In your opinion and based on scientific evidence, what are the main challenges that should be addressed to ensure the sustainability of intensive agriculture in Almeria in the next decades? Identify and list at least three
  Challenge 1 ……………………………………………………………………………………….
  Challenge 2 ……………………………………………………………………………………….
  Challenge 3 ……………………………………………………………………………………….
4. Are you interested in participating as collaborator of an academic publication as result of this workshop?
  Yes ……………………………………………………………………………….…………………
  No ……………………………………………………………………….………………………….

## Appendix B

**Table A1:** Workshop Agenda.

08:45–09:00	Reception
09:00–09:10	Workshop Inauguration—Vice-rector of Research, Development and Innovation
09:10–09:40	Introduction
09:40–10:40	Presentation of participants and exposition of individual challenges
10:40–11:00	Coffee Break
11:00–12:00	Collective definition of 3 priority challenges by the working groups
12:00–12:30	Plenary 1: Exhibitions
12:30–13.30	Opportunities and barriers to meet the challenges posed
13:30–14:00	Plenary 2: Exhibitions
14.00–15:00	Lunch
15:00–16:00	Actions to meet the challenges (next steps)
16:00–17:00	Plenary 3: Exhibitions
17:00–18:00	Synthesis of results and Workshop Closing
